# Relationship between caregiver burden and family resilience among Chinese caregivers of people with dementia: the mediating role of mutuality

**DOI:** 10.3389/fpsyt.2026.1724514

**Published:** 2026-02-10

**Authors:** Mengli Yang, Jiewen Zhang, Xiao Liu, Yanming Ma, Qiuhuan Jiang, Shuai Chen, Shuang Zhang

**Affiliations:** 1 Department of Neurology, Henan Provincial People’s Hospital, Henan Provincial Key Medicine Laboratory of Nursing, Zhengzhou University People’s Hospital, Zhengzhou, China; 2 Department of Neurology, Henan Provincial People’s Hospital, Zhengzhou University People’s Hospital, Zhengzhou, China; 3 School of English Studies, Tianjin Foreign Studies University, Tianjin, China; 4 Henan Provincial People’s Hospital, Henan Provincial Key Medicine Laboratory of Nursing, Zhengzhou University People’s Hospital, Zhengzhou, China

**Keywords:** caregiver burden, dementia, family caregivers, family resilience, mutuality

## Abstract

**Background:**

Caregiving for people with dementia imposes significant psychological and physical burdens on family caregivers, which may affect overall family functioning. This study aimed to examine whether mutuality statistically mediates the relationship between caregiver burden and family resilience among Chinese dementia caregivers.

**Methods:**

This cross-sectional research was conducted from October 2022 to December 2023 across two tertiary hospitals in Henan Province, China. A total of 296 family caregivers of people with dementia participated in the study. Caregivers completed the Chinese versions of the Zarit Burden Interview, the Mutuality Scale, and the Family Resilience Assessment Scale. Pearson correlations were used to examine associations between caregiver burden, mutuality, and family resilience. Structural equation modeling was performed in AMOS 24.0 to assess the mediating role of mutuality.

**Results:**

Caregiver burden was negatively associated with mutuality (*p* <.01) and family resilience (*p* <.01). Mutuality (total score) and its four dimensions demonstrated positive correlations with family resilience (*p* <.01). Furthermore, mutuality significantly mediated the relationship between caregiver burden and family resilience (*p* <.01), with a mediating effect of 39.0%.

**Conclusions:**

This study suggests that mutuality may be an important relational process associated with both caregiver burden and family resilience in dementia caregiving. Public health interventions may benefit from strengthening the caregiver-care recipient relationship to reduce psychological burden and enhance family resilience. Community-based programs and caregiver support initiatives that foster mutual understanding and positive interactions may be particularly effective in promoting the health and well-being of both caregivers and patients.

## Introduction

China currently has the highest number of people with dementia, posing a huge challenge to both the public and healthcare system (1). According to the model-based projection of dementia prevalence, the number of dementia patients in China was 16.25 million in 2020, expected to nearly triple to 48.98 million by 2050 (2). Due to the complex and irreversible nature of dementia, the role of family caregivers becomes crucial. In China, about 80% of people with dementia primarily receive care at home from family members (3). More than 11 million family members and other unpaid caregivers provided an estimated 18.4 billion hours of care to people with dementia in 2023 (4). The long-term continuous care and the rapidly growing demand for care services have imposed a heavy burden on family caregivers (5).

Caregiver burden is the multidimensional stress of physical, psychological, social, and financial aspects that caregivers endure while caring for their relative (6). As dementia progresses, patients increasingly depend on caregivers for daily activities. Due to the lack of professional competence, social support and help, family members face heavy psychophysical burden (7, 8). Compared with caregivers of other illnesses, family caregivers of people with dementia suffer a significantly higher level of caregiver burden (9, 10). According to the three-year longitudinal study by Connors et al. (11), a large proportion of family caregivers experienced a greater caregiver burden as the duration of care increases. The progressively increasing caregiver burden is associated with poorer physical health, psychological well-being, and quality of life for both dementia patients and their caregivers (12–14). It is also linked to strained family relationships, diminished family adaptability, and a compromised overall structure and functionality of the family unit (15). Therefore, identifying factors that can buffer these negative effects and sustain positive family functioning is imperative.

In this context, family resilience plays a significant role. It refers to the ability of family members to recover from misfortune, threat, trauma or crisis (16). As a dominant force within the family, resilience helps members collectively address and adapt to pressures, crises, and adversities, promoting mental health and sustaining family functionality (17–19). Studies have shown that long-term caregiver burden correlates with family problems, conflicts, and diminished family relationships, and may also be associated with lower levels of family resilience (20, 21). Furthermore, chronic stress tends to amplify the negative impact of burden on family resilience (22). Caregivers with higher family resilience are better able to utilize internal and external resources to manage stressors, which may contribute to more favorable health outcomes (23). These findings underscore the need to examine potential relational processes that may help explain the interplay between caregiver burden and family resilience under the cross-sectional design.

One promising relational factor is mutuality, defined as the perceived quality of the dyadic caregiver-care recipient relationship, characterized by shared experiences and reciprocity (24). Mutuality encompasses four dimensions: love and affection, shared pleasant activities, reciprocity, and shared values (25). Unlike general social support, which focuses on external networks and resources, mutuality emphasizes the emotional and relational quality within the caregiver-care recipient dyad (24). This makes it particularly relevant to family resilience by fostering emotional bonds and shared responsibilities that support coping with caregiving stress. Theoretically, mutuality can be understood within both McCubbin’s Resiliency Model of Family Stress Adjustment and Adaptation (RMFAA) and the Stress Process Model (26–28). In RMFAA, mutuality is viewed as an internal family resource that may help the family adapt to caregiving stressors, while the Stress Process Model highlights relational resources like mutuality as critical to family functioning under stress. Mutuality is a protective factor in caregiving and is associated with the positive outcomes for caregivers in the context of chronic diseases (29). Recently, mutuality within dementia patient-caregiver dyads has gained increasing attention. A prospective cohort study indicated that mutuality may be a useful predictor of psychosocial functioning among people with dementia and their caregivers (30). Thus, mutuality serves as a crucial interpersonal process that could theoretically link the experience of caregiver burden with the development or maintenance of family resilience.

In the Chinese cultural context, caregiving is deeply influenced by traditional values such as filial piety, collectivism, and intergenerational reciprocity. Filial piety emphasizes the moral duty of children to care for their elderly parents, deeply influencing caregiving practices in Chinese families (31). Collectivist family values highlight the importance of family cohesion, mutual support, and shared responsibilities, which are integral to the quality of caregiver-care recipient interactions (32). Intergenerational reciprocity, another key aspect, involves mutual support between generations (33). Within this cultural framework, mutuality plays a significant role in caregiving, involving emotional connection, shared responsibilities, and interdependence between caregivers and care recipients (25). This relational quality plays a role in how caregiving stress is managed and how families adapt to challenges, which may relate to family resilience (34, 35). Therefore, in the context of Chinese caregiving, mutuality functions not only as an individual resource but also as a collective family asset, aiding families in managing caregiving challenges and maintaining family harmony and functionality.

Although prior studies have reported pairwise correlations among caregiver burden, mutuality, and family resilience (36–39), the role of mutuality needs further clarification. Mutuality was selected as the focal relational process because it captures the dyadic quality of the core caregiving relationship, which is distinct from individual-level appraisals (e.g., coping, self-efficacy) and external resources (e.g., social support). From a theoretical perspective, both the RMFAA and the Stress Process Model conceptualize relational and psychosocial resources as intervening processes linking stressors to family-level outcomes (26–28). Accordingly, mutuality is analytically positioned between caregiver burden and family resilience, representing a relational process through which caregiving stress may be associated with broader family adaptation, rather than being treated as an antecedent trait or an outcome. At the same time, we acknowledge that alternative conceptual pathways are theoretically plausible, including moderation effects or reverse directional associations (e.g., family resilience shaping mutuality, or mutuality being related to caregiver burden). Given the cross-sectional nature of the data, the proposed mediation model does not imply causality but reflects a theory-informed framework aimed at clarifying how caregiver burden, relational processes, and family resilience are interconnected among Chinese dementia caregivers.

While prior studies, such as Shao et al. (40), have demonstrated associations among mutuality, caregiver burden, and family resilience in cancer caregiving contexts, these findings cannot be readily generalized to dementia caregiving, which is characterized by progressive cognitive decline and long-term relational strain. Existing research has largely focused on pairwise correlations, leaving the underlying relational mechanisms insufficiently examined, particularly the role of mutuality as an intervening process linking caregiving stress to family-level adaptation. Moreover, mutuality has rarely been situated within an integrated theoretical framework that accounts for culturally embedded caregiving norms, such as filial piety and intergenerational reciprocity, which are central to Chinese family caregiving. Addressing these gaps, the present study advances theory by positioning mutuality as a relational mediator within an integrated framework combining the RMFAA and the Stress Process Model. This relationship-centered approach extends existing resilience theories beyond individual stressors or external resources to highlight dyadic relational quality as a core pathway shaping family resilience. By doing so, the study contributes to the global dementia caregiving literature through a culturally grounded model that clarifies how relational processes operate in Chinese families and provides actionable insights for interventions aimed at strengthening family resilience in dementia care.

Thus, this study aims to explore the relationship between caregiver burden, mutuality, and family resilience, and verify the mediating role of mutuality between caregiver burden and family resilience among Chinese dementia caregivers.

### Conceptual framework

**Figure 1:**
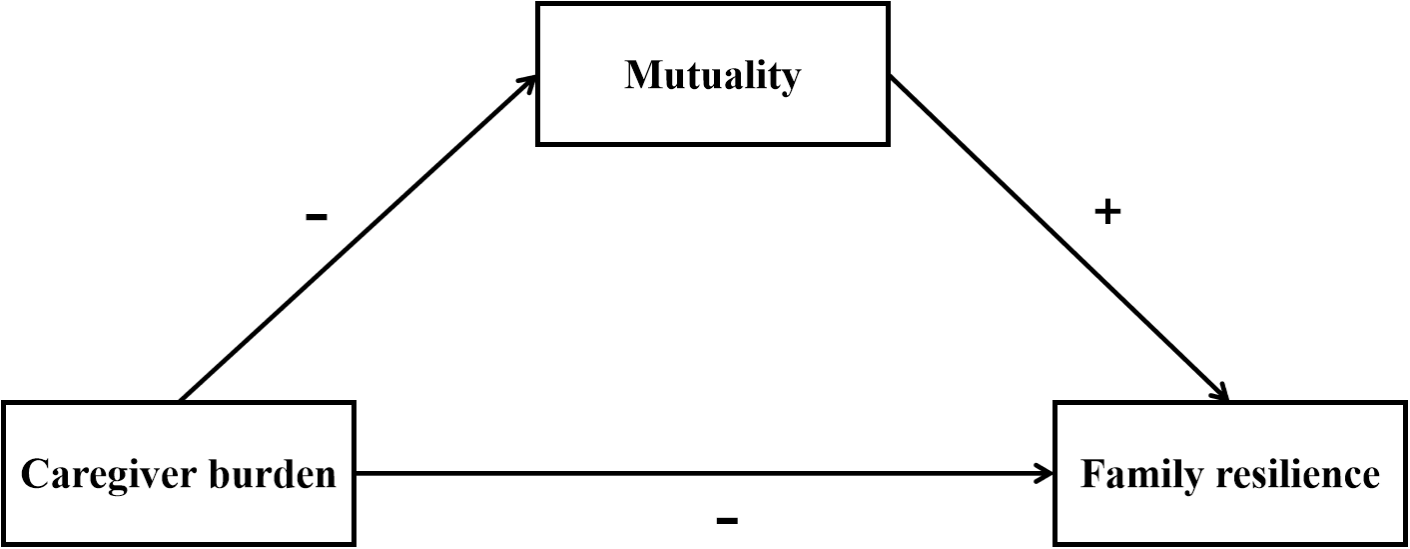
Hypothesized conceptual framework.

The RMFAA mainly focuses on the adjustment and adaptation process of families when facing stressful events, emphasizing the use of internal and external resources to maintain resilience (26). In this framework, caregiver burden is conceptualized as a stressor, mutuality as an internal family resource, and family resilience as an adaptation outcome. Complementarily, the Stress Process Model, widely applied in dementia caregiving research, highlights the influence of various stressors on caregiver outcomes and the mediating role of psychosocial resources, with mutuality functioning as a relational mediator (27, 28). By integrating these two frameworks, the conceptual model clarifies how caregiver burden (stressor), mutuality (internal relational resource/mediator), and family resilience (adaptation outcome) are interconnected. The integration is complementary: RMFAA provides a family-centered perspective on resource mobilization and adaptation, while the Stress Process Model emphasizes the relational and psychosocial pathways linking caregiving stress to family outcomes. Together, these models provide stronger explanatory power than either alone, capturing both family resource mobilization and relational-psychosocial processes. Based on this integrated framework, we hypothesized that higher caregiver burden is associated with lower family resilience, with mutuality potentially mediating this association. The conceptual framework of this study is presented in **Figure 1**.

Based on the conceptual framework, we proposed the following hypotheses:

Hypothesis1 Caregiver burden is expected to be negatively correlated with family resilience.
Hypothesis2 Caregiver burden is expected to be negatively related to mutuality.
Hypothesis3 Mutuality is expected to be positively correlated with family resilience.
Hypothesis4 Mutuality is expected to mediate the relationship between caregiver burden and family resilience.

## Methods

### Study design

This was a cross-sectional study using a convenience sampling method. The study was reported in accordance with the Strengthening the Reporting of Observational Studies in Epidemiology (STROBE) guidelines (41).

### Participants

This study was conducted from October 2022 to December 2023 in Henan Province, China. Family caregivers of dementia patients were recruited from the neurology department of two tertiary hospitals. The inclusion criteria were (1): the care recipient was diagnosed with dementia by a psychiatrist or neurologist; (2) aged 18 years or older; (3) assumed the primary responsibility in caring for patients with dementia; (4) provide care time > 3 months; (5) able to communicate and interact normally; (6) able to provide informed consent and voluntary participation in this research. The exclusion criteria were: (1) had serious physical illnesses; (2) had severe cognitive impairment or mental disorders; (3) employed professional caregivers.

Eligible caregivers were identified through medical records and referrals from neurologists and nursing staff in the neurology departments. Trained investigators approached potential participants during inpatient hospitalization and provided a brief explanation of the study. All eligible caregivers during the study period were invited to participate. During the study period, a total of 320 caregivers were approached, of whom 310 agreed to participate and completed the questionnaire, yielding a response rate of 96.9%. The primary reasons for refusal included lack of time (n=2) and unwillingness to disclose personal information (n=8). Among the 310 participants surveyed, 14 were excluded due to incomplete or inconsistent data. Thus, 296 valid responses were included in the final analysis.

### Sample size

An *a priori* power analysis was conducted using Monte Carlo Power Analysis for Indirect Effects to determine the required sample size (42). The target power (1-β) was set at 0.90, with a 95% confidence level (α = .05). The sample size was calculated to be 149 based on the pre-experimental data, with the following standardized path coefficients: the path from caregiver burden to mutuality (a) = -.29, the path from mutuality to family resilience (b) = .29, and the direct path from caregiver burden to family resilience (c’) = -.30. Considering a wastage rate of 20%, the number of participants required was 187.

### Procedure and ethical considerations

Prior to initiating the study, ethical approval was obtained from the Medical Ethics Committee of Henan Provincial People’s Hospital (Approval No: 2020-076). This study was conducted in accordance with the Declaration of Helsinki. All participants received a detailed explanation of the study purpose, procedures, and their rights. Written informed consent was obtained before participation. Participants were informed that they could withdraw from the study at any time without any consequences. No monetary or material compensation was provided for participation. Questionnaires were completed either independently by participants or with assistance from trained researchers if necessary. To maintain anonymity, caregiver and patient data were coded, and identifying information was not linked to the survey responses. Psychological risks were minimized by allowing participants to skip any question that made them uncomfortable and by providing access to psychological support services if needed. All collected data were stored securely and treated as strictly confidential.

### Measures

#### Sociodemographic and clinical characteristics

Participant characteristics were collected using a self-designed questionnaire. Sociodemographic information from dementia patients included age, gender, marital status, and education. The caregivers’ sociodemographic data included age, gender, marital status, education, job, relationship to the patient, living with patients, duration of caregiving, and daily caregiving hours. Clinical characteristics of patients, including duration of illness, type of dementia, severity of disease, and number of comorbidities, were collected from medical records. The severity of disease was assessed using the Clinical Dementia Rating Scale, which was obtained from the patients’ medical records with their doctor’s permission.

### Zarit Burden Interview

The Chinese version of 22-item Zarit Burden Interview (ZBI) scale was used to assess the family caregiver burden in the present study (43). The scale consisted of two dimensions: personal burden and responsibility burden. A five-point Likert scale was used, with each item scoring from 0 (never) to 4 (nearly always) and a total score ranging from 0 to 88. The total score was used to identify the caregiver burden, with higher scores reflecting higher levels of caregiver burden. The Chinese version scale showed good reliability and validity in research on family caregivers with dementia (Cronbach’s alpha= .942) (44). In our study, the Cronbach’s alpha of this scale was.938.

### Mutuality scale

The Chinese version of 15-item Mutuality Scale (MS) was used to assess caregiver mutuality (45). The scale contained four dimensions: pleasurable activities, love and affection, shared values, and reciprocity. Each item was answered using a five-point Likert scale ranging from 0 (not at all) to 4 (a great deal). The total score for the scale was obtained by the mean value of all the individual items’ scores (25, 45). Higher scores indicated better quality of relationship between the care dyads. The reliability and validity of the scale have been supported in a previous study among caregivers of patients with dementia (Cronbach’s alpha= .94) (45). The Cronbach’s alpha of this scale was recorded as.913 in this study.

### Family Resilience Assessment Scale-Chinese Version

We used the 32-item Chinese version of Family Resilience Assessment Scale (FRAS-C) to measure caregivers’ family resilience (46). The scale composed of three subscales: Maintaining a Positive Outlook (MPO), Family Communication and Problem Solving (FCPS), and Utilizing Social Resources (USR). Each item was rated on a four-point Likert scale from 1 (strongly disagree) to 4 (strongly agree). The scale scores ranged from 32 to 128, with higher scores indicating stronger family resilience. The Chinese version scale demonstrated adequate internal consistency, with a Cronbach’s alpha of.958 (47). In the current study, the Cronbach’s alpha of this scale was.969.

### Data analysis

This study used IBM SPSS version 26.0 to analyze the data. Descriptive statistics were used to describe the participant sociodemographic characteristics and levels of caregiver burden, mutuality, and family resilience. Continuous variables were checked for normality test and presented as mean ± standard deviation, categorical variables were described as frequencies and percentages. We adopted Pearson’s correlation analysis to test the bivariate relationships among caregiver burden, mutuality, and family resilience.

To examine the hypothesized relationships among the main study variables, IBM SPSS AMOS 24.0 was employed to construct structural equation modeling using the maximum likelihood estimation method. Prior to testing the structural relationships, a measurement model was specified and evaluated using confirmatory factor analysis (CFA). The measurement model included three latent variables: caregiver burden (two indicators: personal burden and responsibility burden), mutuality (four indicators: pleasurable activities, love and affection, shared values, and reciprocity), and family resilience (three indicators: MPO, FCPS, and USR). The model fit indices included Chi-square/degrees of freedom (*X^2^/df*), Root Mean Square Error of Approximation (RMSEA), Normed Fit Index (NFI), Incremental Fit Index (IFI), Tucker-Lewis Index (TLI), and Cumulative Fit Index (CFI). An acceptable model fit was indicated by *X^2^/df* < 3, RMSEA<.08, and incremental fit indices (NFI, IFI, TLI, and CFI) greater than.90, consistent with established guidelines (48). Composite reliability and average variance extracted (AVE) were calculated for each latent construct to assess internal consistency and convergent validity. The bootstrap method was used to test the mediating effect in the structural model. The bias-corrected 95% confidence interval (CI) was calculated via a 5000-sample bootstrap procedure. A mediation effect was deemed statistically significant if the 95% CI did not include zero. Statistical significance was set at *p* <.05.

## Results

### Sample characteristics

Participants’ sociodemographic and clinical characteristics are described in **Table 1**. The mean age of people with dementia was 70.86 (SD = 8.15), ranging from 60 to 94. More than half of the patients were male (51.4%), and had a moderate severity of dementia (64.2%). The type of dementia was mainly Alzheimer’s disease in 116 cases (39.2%). The duration of illness was varied: 42 cases (14.2%) < 1 year, 155 cases (52.4%) from 1 year to 3 years, and 99 cases (33.4%) > 3 years. The mean age of caregivers was 52.75 (SD = 13.11), ranging from 28 to 82. The caregivers were predominantly female (61.8%), and mostly living with their patients (79.7%). At least 81.1% of the caregivers reported middle school education or higher. Over half of the carers were children of the patients (52.0%), employed (53.7%), and married (95.9%). For detailed information, refer to **Table 1**.

**Table 1:** Sociodemographic and clinical characteristics of study participants (N = 296).

Characteristic	n (%)/mean ± SD	Characteristic	n (%)/mean ± SD
People with dementia		Caregivers	
Age (years), Mean ± SD	70.86 ± 8.15	Age (years), Mean ± SD	52.75 ± 13.11
Gender		Gender	
Male	152 (51.4)	Male	113 (38.2)
Female	144 (48.6)	Female	183 (61.8)
Marital status		Marital status	
Unmarried	21 (7.1)	Unmarried	12 (4.1)
Married	275 (92.9)	Married	284 (95.9)
Education level		Education level	
Primary school or below	144 (48.6)	Primary school or below	56 (18.9)
Middle school	59 (19.9)	Middle school	88 (29.7)
High school	51 (17.3)	High school	66 (22.3)
College or above	42 (14.2)	College or above	86 (29.1)
Type of dementia		Job	
Alzheimer’s disease	116 (39.2)	Unemployed or retired	137 (46.3)
Vascular dementia	90 (30.4)	Employed	159 (53.7)
Mixed dementia	51 (17.2)	Relationship with patients	
Other	39 (13.2)	Spouse	126 (42.6)
Severity of disease		Children	154 (52.0)
Mild	73 (24.7)	Other family members	16 (5.4)
Moderate	190 (64.2)	Living with patients	
Severe	33 (11.1)	Yes	236 (79.7)
Duration of illness (years)		No	60 (20.3)
<1	42 (14.2)	Duration of caregiving (years)	
1~3	155 (52.4)	<1	42 (14.2)
>3	99 (33.4)	1~3	179 (60.5)
Number of comorbidities		>3	75 (25.3)
<3	229 (77.4)	Care time per day (hours)	
≥h	67 (22.6)	<8	100 (33.8)
		8~12	69 (23.3)
		>12	127 (42.9)

### Descriptive statistics and correlations of caregiver burden, mutuality, and family resilience

**Table 2** shows the means, standard deviations, and correlations of all the study variables. There was a significant correlation between caregiver burden, mutuality, and family resilience. Caregiver burden was negatively associated with mutuality (*r* = -.704, *p* <.01) and family resilience (*r* = -.626, *p* <.01), indicating that higher levels of burden were linked to lower perceived mutuality and lower family resilience. In contrast, mutuality (total score) and its four dimensions were positively correlated with family resilience, with correlation coefficients ranging from.563 to.634 (all *p* <.01), suggesting that higher mutuality is consistently associated with stronger family resilience.

**Table 2:** Descriptive statistics and correlations of all variables (N = 296).

Variable	Mean	SD	1	2	3	4	5	6	7	8	9	10	11	12
1. Caregiver burden	32.43	17.90	1											
2. Personal burden	18.84	9.26	.965^**^	1										
3. Responsibility burden	7.62	5.80	.913^**^	.793^**^	1									
4. Mutuality	2.77	.35	-.704^**^	-.683^**^	-.644^**^	1								
5. pleasurable activities	3.07	.40	-.694^**^	-.668^**^	-.640^**^	.926^**^	1							
6. Love and affection	3.37	.46	-.642^**^	-.619^**^	-.598^**^	.909^**^	.811^**^	1						
7. Shared values	2.42	.43	-.651^**^	-.641^**^	-.581^**^	.819^**^	.756^**^	.690^**^	1					
8. Reciprocity	2.39	.34	-.579^**^	-.565^**^	-.527^**^	.917^**^	.760^**^	.764^**^	.651^**^	1				
9. Family resilience	99.91	14.45	-.626^**^	-.603^**^	-.590^**^	.634^**^	.571^**^	.588^**^	.572^**^	.563^**^	1			
10. MPO	19.13	2.80	-.583^**^	-.554^**^	-.572^**^	.552^**^	.488^**^	.518^**^	.527^**^	.483^**^	.916^**^	1		
11. FCPS	71.56	11.03	-.624^**^	-.601^**^	-.584^**^	.645^**^	.581^**^	.599^**^	.578^**^	.574^**^	.991^**^	.868^**^	1	
12. USR	9.22	1.35	-.396^**^	-.389^**^	-.360^**^	.368^**^	.355^**^	.323^**^	.300^**^	.336^**^	.703^**^	.635^**^	.637^**^	1

**p <.01.

FCPS, family communication and problem solving; USR, utilizing social resources; MPO, maintaining a positive outlook.

### Measurement model

A confirmatory factor analysis (CFA) was conducted to evaluate the measurement model prior to testing the structural relationships. The measurement model consisted of three latent variables: caregiver burden (two indicators), mutuality (four indicators), and family resilience (three indicators). The results indicated an acceptable fit of the measurement model to the data (*X^2^/df* = 2.496, RMSEA = .071, NFI = .973, IFI = .984, TLI = .975, and CFI = .983). All observed indicators loaded significantly onto their respective latent constructs (*p* <.001), with standardized factor loadings ranging from.668 to.963 (see **Table 3**). The composite reliability for each latent construct ranged from.885 to.920, and average variance extracted ranged from.730 to.793, demonstrating strong internal consistency and convergent validity. No *post hoc* modifications were applied to the model, and no correlated errors were specified between the indicators to maintain the theoretical integrity and simplicity of the measurement model. The latent variable modeling approach was chosen over composite modeling to account for measurement error explicitly, providing more accurate estimates of the relationships among the latent constructs.

**Table 3:** Standardized factor loadings and convergent validity.

Latent variable	Observed indicator	Estimate	Composite reliability	Average variance extracted
Caregiver burden	Personal burden	.909	.885	.793
	Responsibility burden	.872		
Mutuality	pleasurable activities	.919	.920	.743
	Love and affection	.884		
	Shared values	.810		
	Reciprocity	.831		
Family resilience	MPO	.903	.888	.730
	FCPS	.963		
	USR	.668		

FCPS, family communication and problem solving; USR, utilizing social resources; MPO, maintaining a positive outlook.

### Mediation analysis of mutuality in the relationship between caregiver burden and family resilience

**Figure 2:**
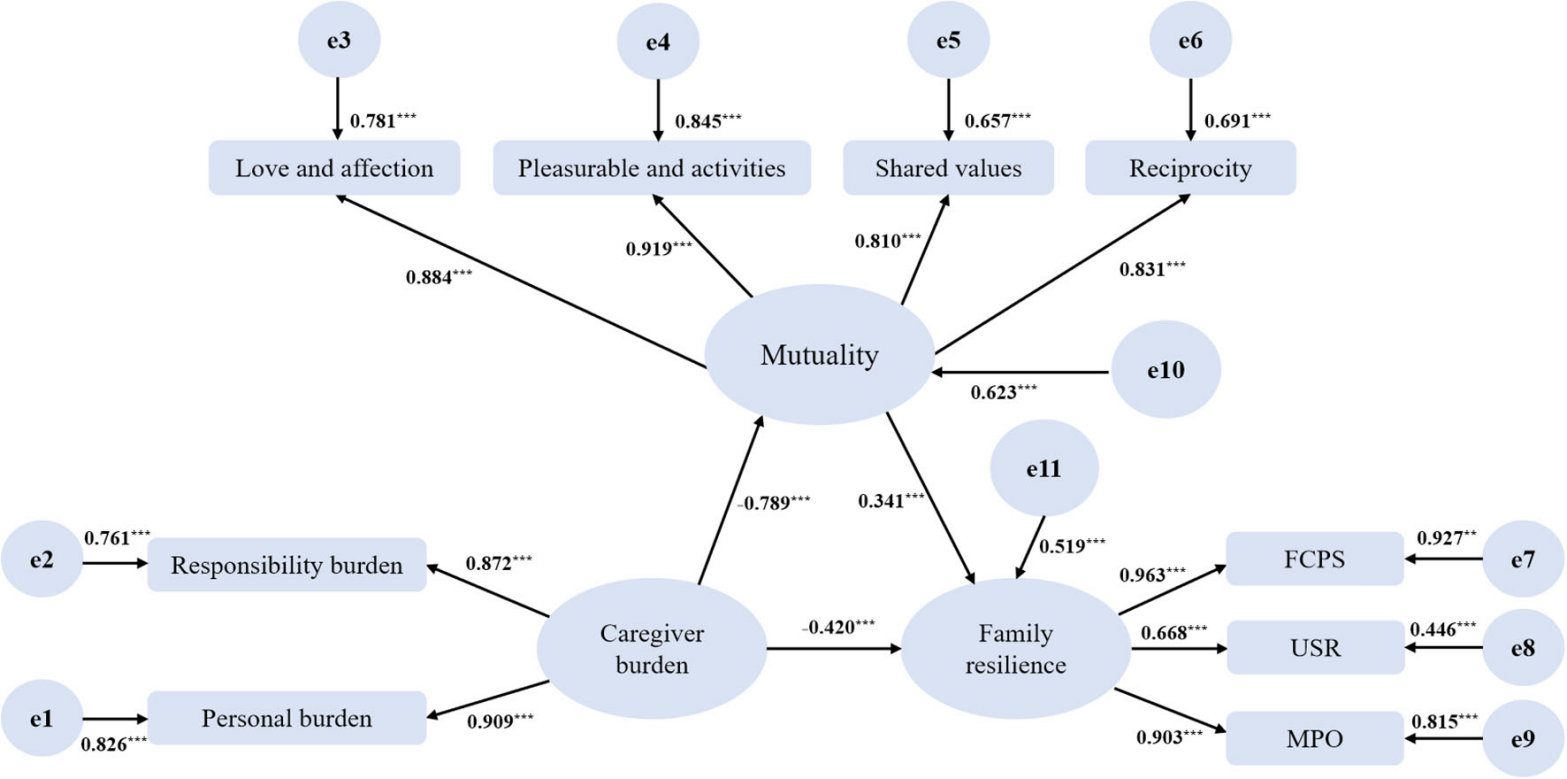
Model of the associations between caregiver burden, mutuality, and family resilience. **p <.01, ***p <.001. Values on paths are path coefficients (standardized βs). The ‘e’ labels (e1-e11) denote residual/error terms: e1-e9 are measurement errors for the indicators, and e10-e11 are disturbances (residuals) for the latent variables Mutuality and Family Resilience. FCPS, family communication and problem solving; USR, utilizing social resources; MPO, maintaining a positive outlook.

Based on the measurement model results, a structural equation model was specified to examine the associations among caregiver burden, mutuality, and family resilience within a mediation analysis framework. In this model, caregiver burden was treated as the independent variable, mutuality as the mediating variable, and family resilience as the outcome variable. This model matched well to the data, with its fit indices aligning with those of the measurement model: *X^2^/df* = 2.496, RMSEA = .071, NFI = .973, IFI = .984, TLI = .975, and CFI = .983, indicating that the proposed associations among latent variables were consistent with the measurement structure. As shown in **Table 4**, caregiver burden was negatively associated with both mutuality (*β* = -.789, *p* <.001) and family resilience (*β* = -.420, *p* <.001). Conversely, mutuality was positively associated with family resilience (*β* = .341, *p* <.001). Importantly, the squared multiple correlations (R²) indicated that the model explained 62.3% of the variance in mutuality and 51.9% of the variance in family resilience, with the remaining variance captured by the residual terms (e10 and e11), representing unexplained variance. As presented in **Table 5**, mutuality revealed a significant indirect association between caregiver burden and family resilience. The total effect of caregiver burden on family resilience was significant (effect size = -.689, 95% CI: -.756, -.608). The direct effect of caregiver burden on family resilience was also significant (effect size = -.420, 95% CI: -.600, -.236). Furthermore, a significant indirect effect via mutuality was found (effect size = -.269, 95% CI: -.408, -.120), accounting for 39.0% of the total effect. This suggests a moderate indirect effect of caregiver burden on family resilience through mutuality, indicating that mutuality plays a substantial role in explaining the relationship between caregiver burden and family resilience. The tested model is shown in **Figure 2**.

**Table 4:** Standardized estimation of each path in structural equation model.

Path	*β*	Estimate	SE	CR	*P*
Caregiver burden → Mutuality	-.789	-.038	.003	-14.334	<.001
Caregiver burden → Family resilience	-.420	-.530	.112	-4.734	<.001
Mutuality → Family resilience	.341	8.971	2.269	3.954	<.001

*β*, standardized regression coefficient; SE, standard error; CR, critical ratios.

**Table 5:** Total, direct, and indirect effects in the tested mediation model.

Effects	Effect size	SE	Bootstrapping (BC 95% CI)		P	Effect (%)
			Lower	Upper		
Direct effect	-.420	.092	-.600	-.236	.001	61.0
Indirect effect	-.269	.073	-.408	-.120	.002	39.0
Total effect	-.689	.038	-.756	-.608	<.001	100.0

SE, standard error; CI, confidence interval.

## Discussion

To the best of our knowledge, this study is the first to investigate the relationships among caregiver burden, mutuality and family resilience in the context of Chinese families caring for dementia patients. The findings support all proposed hypotheses and highlight a central insight: mutuality, the quality of the caregiver-care recipient relationship, plays a pivotal role in the pathway between caregiver burden and family resilience. This suggests that fostering family resilience may benefit more from targeting relationship quality than solely addressing caregiver burden.

Regarding the level of family resilience, the observed scores in our sample were higher than those reported for caregivers of stroke and breast cancer patients (49, 50). These differences may be due to most dementia patients in this study presenting with mild to moderate symptoms and relatively short disease progression. In addition, 74.7% of caregivers had provided care for less than three years, which may correspond to fewer accumulated negative emotions and relatively higher psychological resilience levels (51). These characteristics likely allowed families to mobilize resources and maintain adaptive functioning, illustrating that family resilience is dynamic and influenced by caregiving stage and context. Future research could examine these factors more systematically, and healthcare providers may explore interventions that focus on supporting family resilience by drawing on these adaptive capacities.

The negative association observed between caregiver burden and family resilience may reflect how sustained caregiving demands interact with family system processes, potentially challenging family structure and daily functioning (52). This aligns with findings reported by Jia et al. (44), suggesting that higher levels of caregiver burden tend to coexist with lower levels of family resilience within long-term caregiving contexts. Similarly, greater caregiver burden appears to be associated with lower mutuality, consistent with Shao et al. (40). Previous review indicated that caregiver-care receiver mutuality is closely linked to emotional well-being (29). Caregivers with higher burden often report more negative emotions and lower intimacy within caregiving dyads (53), providing context for the observed association. The positive association between mutuality and family resilience highlights the role of mutuality as a key relational resource within family systems facing sustained caregiving demands (39, 40). Studies have shown that improving mutuality is linked to better communication and interaction, which may help families cope with challenges and strengthen family adaptability and resilience (40, 54). Together, these findings underscore the importance of relational quality in understanding and supporting family resilience in the context of dementia caregiving.

The key finding of the current study was that mutuality was a significant mediator in the association between caregiver burden and family resilience, supporting hypothesis 4. This mediation reflects a statistical association rather than evidence of an underlying causal mechanism, given the cross-sectional nature of the study. From an integrated theoretical perspective, this indirect pathway is congruent with and provides an empirical basis for integrating the RMFAA and the Stress Process Model. Caregiver burden can be considered a stressor that is linked to variations in relational resources, such as mutuality. Mutuality may serve as a relational resource that could help caregivers manage caregiving-related stress and be associated with family resilience, although the underlying psychological mechanisms were not directly measured in this study. Through this empirical link, the RMFAA, emphasizing family resource mobilization and adaptation, and the Stress Process Model, highlighting emotional and relational factors in coping with stress, are synthesized into a coherent framework for understanding how caregiver burden, mutuality, and family resilience are interconnected (26–28). While mutuality was identified as a statistically significant mediator, this should be interpreted as an association rather than an established psychological mechanism. Further research is needed to explore alternative explanations for the observed associations, including the influence of external support resources, individual coping styles, and to examine whether the mediating role of mutuality differs across cultural contexts or stages of dementia.

In the context of Chinese caregiving, the observed mediating role of mutuality may be particularly meaningful due to culturally shaped family dynamics. Filial piety, family harmony, and collectivist values strongly emphasize caregiving as a moral and relational responsibility (31–33). Within this framework, mutuality may function as a central relational resource, supporting family cohesion, shared identity, and collective responsibility. High caregiver burden may place strain on this relationship, while strong mutuality may help families navigate caregiving challenges and maintain resilience. These findings suggest that the pathway from caregiver burden to family resilience via mutuality carries distinct cultural significance, highlighting the need for culturally informed interpretations and interventions that leverage relational strengths in Chinese families.

### Implications

Based on the findings of this study, several specific recommendations are proposed to enhance family resilience and support caregivers of individuals with dementia. First, It is necessary for healthcare professionals to regularly assess caregiver burden and identify needs related to physical, emotional, social, and communication support. Interventions such as digital psychological interventions (55), psychoeducation, and multi-component programs (56) could be implemented to reduce caregiver burden. Second, enhancing mutuality within the caregiver-care recipient relationship is a key pathway to increasing family resilience. Interventions may focus on dyadic sensory art therapies, as well as psychoeducation with active skills training designed for both caregivers and care recipients to strengthen their relationship quality. Third, family support systems are crucial in enhancing family resilience. Healthcare providers could implement holistic family intervention programs, such as psychoeducation, mindfulness, or cognitive-behavioral approaches (57), group-based family resilience intervention (58), to help family members collaborate and support each other in caregiving tasks. Lastly, considering the cultural context is essential for developing effective interventions in Chinese families. The emphasis on filial piety and collectivist values in Chinese caregiving contexts suggests that interventions tailored to strengthen relational cohesion in caregiving dyads could yield significant benefits. Programs designed with these cultural norms in mind may further enhance mutuality and family resilience.

### Limitations

This research has several limitations. First, the participants were recruited from only two hospitals in a single area (Zhengzhou, Henan Province, China), limiting the generalizability of the current results. Further studies should include multiple regions, more and different levels of hospitals to enhance the representative of the results. Second, this cross-sectional study cannot establish causality among the three variables, and longitudinal or interventional studies are recommended to explore these associations with greater certainty. Third, as the data were collected through self-report questionnaires, response bias may exist, although the instruments have been proven reliable and effective in previous studies. Fourth, we collected caregiver characteristics only, interaction effects between patients and caregivers in relationships among the three variables could not be examined. Incorporating patients’ perspectives would allow a more comprehensive exploration of dyadic relationships. In addition, although we treated mutuality as a mediator between caregiver burden and family resilience, the cross-sectional design limits causal interpretation of this pathway. Moreover, potential confounding factors, such as external support resources, patient characteristics, and cultural influences, were not included in the model. Future studies could consider these variables to refine the theoretical framework. Nevertheless, these findings highlight key targets that may be considered for caregiver support and family resilience interventions in dementia care.

## Conclusion

This study identified caregiver burden and mutuality as key factors associated with family resilience among dementia caregivers. Mutuality emerged as a significant intermediary in the association between caregiver burden and family resilience. These findings highlight mutuality, the quality of the caregiver-care recipient relationship, as a pivotal point for public health interventions aimed at supporting family resilience. In terms of practice, attention may be directed toward early screening for relational strain within caregiver-patient dyads during routine dementia care assessments, the development of brief dyad-focused interventions aimed at strengthening communication, shared understanding, and emotional connection, and the integration of such relational support into existing community-based care programs to enhance accessibility and sustainability. Prioritizing the quality of the caregiving relationship may offer a meaningful pathway for supporting families as they navigate the challenges of dementia care. 

## Data Availability

The original contributions presented in the study are included in the article/supplementary material. Further inquiries can be directed to the corresponding author.
